# Detecting anomalies from liquid transfer videos in automated laboratory setting

**DOI:** 10.3389/fmolb.2023.1147514

**Published:** 2023-05-04

**Authors:** Najibul Haque Sarker, Zaber Abdul Hakim, Ali Dabouei, Mostofa Rafid Uddin, Zachary Freyberg, Andy MacWilliams, Joshua Kangas, Min Xu

**Affiliations:** ^1^ Computer Science and Engineering Department, Bangladesh University of Engineering and Technology, Dhaka, Bangladesh; ^2^ Computational Biology Department, Carnegie Mellon University, Pittsburgh, PA, United States; ^3^ Department of Psychiatry, University of Pittsburgh, Pittsburgh, PA, United States

**Keywords:** lab automation, video anomaly detection, action recognition, machine learning, feature extraction

## Abstract

In this work, we address the problem of detecting anomalies in a certain laboratory automation setting. At first, we collect video images of liquid transfer in automated laboratory experiments. We mimic the real-world challenges of developing an anomaly detection model by considering two points. First, the size of the collected dataset is set to be relatively small compared to large-scale video datasets. Second, the dataset has a class imbalance problem where the majority of the collected videos are from abnormal events. Consequently, the existing learning-based video anomaly detection methods do not perform well. To this end, we develop a practical human-engineered feature extraction method to detect anomalies from the liquid transfer video images. Our simple yet effective method outperforms state-of-the-art anomaly detection methods with a notable margin. In particular, the proposed method provides 19% and 76% average improvement in AUC and Equal Error Rate, respectively. Our method also quantifies the anomalies and provides significant benefits for deployment in the real-world experimental setting.

## 1 Introduction

Laboratory automation is the integration of machine learning, computer vision, and robotics to automate one aspect or the entire process of a laboratory setting including protocols relying on reagents, tools, and instrument manipulations. With the advent of computation capabilities and artificial intelligence in the last decade, automation has seen a meteoric rise in its applications in laboratories especially as a substitute for repetitive or risky tasks (Felder et al., 1990). The integration of automated components in laboratories is motivated by the necessity of high precision output, reproducibility of experiments, minimized risk/exposure to human operators, and subsequent minimized cost of production through the elimination of manual labor (Bogue, 2012; Holland and Davies, 2020). Proper management of laboratory automation is a stringent requirement in the testing and production process (Saboe, 1995; Holland and Davies, 2020) where defects and failures of the automation component have far-reaching consequences. A significant part of the process is the operators’ abilities to detect these anomalous activities and to intercede when needed. Therefore, anomaly detection in the autonomous laboratory setting plays a pivotal role in ensuring the system’s reliability and safety (Gupta et al., 2018).

Anomaly detection refers to data pattern detection that deviates significantly from the majority of data samples. Pang et al. (2022), Chandola et al. (2009) and Chalapathy and Chawla (2019) state various applications of anomaly detection algorithms ranging from fraud detection, intruder detection, and traffic monitoring to medical anomaly, sensor anomaly, and robotics behavior anomaly. All of these applications range from a variety of data mediums among which automatic anomaly detection in video data has long been a prevalent problem and has drawn a lot of attention from both the research world and industry (Saligrama and Chen, 2012; Zhao et al., 2017; Hao et al., 2022). Live video streams can be used to automatically infer situations of interest by extracting appropriate information from frames (Bebis et al., 2016) and are important in the laboratory setting as a medium for anomaly detection.

The task of liquid transfer in the context of laboratory experiments has profound importance. The mixing of multiple reagents, transfer to and from source and destination containers, and proper handling of the final solution are only some of the applications. Quantitative analysis of such tasks requires proper liquid handling. Automation of such processes enables parallel execution and feedback on a larger scale. This study is based on a novel dataset containing video data from an automated laboratory setting which depicts the automated transfer of liquid reagents via several pipettes from one container to another. This is a repetitive and rudimentary task in the context of biochemical laboratory experiments where precise measurements of the transferred content are required for proper qualitative and quantitative analysis (Betz et al., 2011). Thus, automation of this activity reserves a very important role in the experimentation process but requires complementary detection algorithms to identify anomalous events.

The dataset contains several such anomalous events where the transfer of liquid fails due to different kinds of pipette malfunctions. Additionally, few normal sequences are present where the entire reagent transfer process is executed without any error. Several challenging cases of this dataset are the different types of anomalies introduced and the color variations of the reagents. Furthermore, the dataset provides a limited number of data samples of each type, and consequently, the number of normal sequences is far outnumbered by the combined data of different anomaly sequences. Our work endeavors to find an appropriate solution in order to subvert these challenging cases while limiting the scope of the anomalies related to the task of colored liquid transfer.

Existing anomaly detection solutions have some limitations in the context of this automated laboratory dataset. Adam et al. (2008) and Cheng et al. (2015) employ statistical modeling using features such as optical flows for anomaly detection. But these methods are not generalized enough to be used in the liquid transfer scenario. For example, the optical flow information extracted from the video frames is ineffectual as there are dynamic elements in addition to the region of interest. Deep learning methods are widely popular and have been successfully used for anomaly detection in various settings. These methods include object detection and tracking using trajectory-based methods (Coşar et al., 2016), Convolutional Neural Networks (CNNs) for representation learning (Andrews et al., 2016), and reconstruction methods using Auto-Encoders (Zhao et al., 2017), etc. As these methods automatically extract image features from examples, they require a large number of training samples and a balanced dataset to be properly trained and generalized to all potential scenarios (Alom et al., 2019). The dataset presented here has a class imbalance problem as the normal sequence samples are very few compared to the abnormal sequence samples. Furthermore, the overall number of video samples in the dataset is insufficient for training supervised deep learning methods with reliable cross validation (Fang et al., 2021). Thus, the application of existing supervised deep learning methods in the procured automated reagent transfer dataset is limited. In recognition of these constraints, we instead focus on human-engineered feature extraction methodologies based on observations and assumptions to extract features from the available dataset for the use of machine learning methods to detect anomalies.

In this study, we introduce an anomaly detection algorithm for the automated laboratory setting. Due to the limitations of deep learning approaches in the context of the dataset, we employ custom-made feature extraction methods to develop these algorithms. The algorithm is based on pipette region detection and self-comparison of video frame sequence to quantitatively identify deviation. This is designed specifically for the dataset scenario and overcomes sample size constraints due to the engineered feature extraction property. The algorithm successfully detects all types of colored reagent transfer anomalies present in the dataset. Additionally, extra samples created through manual segmentation from the video data frames are used to extend the dataset reliably.

The layout of this paper is as follows: Section 2 presents a literature review, Section 3 discusses the dataset and its challenges in depth, Section 4 describes the proposed methodologies, Section 5 analyses the results and finally the conclusion is presented in Section 6.

## 2 Related works

Anomaly detection has been developed extensively for a wide range of applications. According to Chalapathy and Chawla (2019), this includes but is not limited to fraud detection, industrial damage detection, medical anomaly detection, video surveillance, etc (Sánchez et al., 2009; Jiang et al., 2011; Arroyo et al., 2015; Maeda et al., 2018; Caruccio et al., 2019; Nawaratne et al., 2019; Wan et al., 2022). Hence, the automatic detection of such anomalies is a popular topic among researchers. In our work, we will focus on detecting anomalies from a video. Though directions explored by researchers to solve such problems are very diversified, the methodologies can be clustered into two major subgroups. Subsection 2.1 illustrates examples from classical hand-crafted feature-based approaches and subsection 2.2 describes some of the modern deep learning-based approaches against this problem.

### 2.1 Classical methods

In the cases where we don’t have access to large labeled data, opting for hand-crafted features and statistical models have been preferred by researchers. Adam et al. (2008) uses monitors in fixed locations around the whole frame and extracts some local feature metrics from them. These features are then used to make a decision. In another work, crowd behavior dynamics are extracted by a social force model (Helbing and Molnar, 1995) and used as the indication factor of anomalies by Mehran et al. (2009). Cheng et al. (2015) extracts interest points from a frame and merges points from different times with Gaussian process regression. These merged points are then compared with samples of an anomalous incident to infer the situation. Pan et al. (2022) proposes a symplectic relevance matrix machine (SRMM) that uses probabilistic models and geometric theory for failure classification.

One important drawback of these types of methods is that these approaches depend on hand-crafted features and losses their generality when a new situation arrives.

### 2.2 Deep learning based methods

Deep learning models, specifically convolutional neural networks are currently used to achieve state-of-the-art performance in a wide range of computer vision problems. This includes image classification (Foret et al., 2020; Brock et al., 2021), object detection (Wang et al., 2022c; Wang et al., 2022a), instance segmentation (Mohan and Valada, 2021; Qiao et al., 2021) etc.

For solving anomaly detection problems, trajectory-based methods (Piciarelli et al., 2008; Coşar et al., 2016) have been proposed as a solution. These methods include two parts. Firstly, the methods detect the objects of interest and secondly, track their trajectory across frames. Deviation in action from normal activities is marked as an anomaly. The performance of this type of procedure depends on both detection and tracking accuracy in this scenario.

A CNN feature extractor can reduce high-dimensional video data into low-dimensional and compact feature vectors and dictionaries. Afterward, these can be passed through some simple classifiers to make a decision (Andrews et al., 2016; Ali et al., 2020; Li et al., 2020; Wang X. et al., 2022).

Reconstruction models represent another deep learning approach that is popular in the detection of anomalous events. In this scenario, a model learns normal patterns while trying to reconstruct frames of normal videos. During inference when an anomalous frame is encountered, the model will generate high reconstruction loss which can clearly indicate the presence of an anomaly. This method has also been supported by many research works (An and Cho, 2015; Zhao et al., 2017; Zenati et al., 2018). One-Class Classification is slightly similar to the reconstruction-based method. The abundance of normal data often leads to using only normal samples while training, thus making the problem into one class classification instead of a binary classification task. Chalapathy et al. (2018) uses this type of solution to address anomaly detection.

There are possibly endless opportunities for using CNNs while addressing a problem like anomaly detection in videos, but for providing satisfactory results, these methods need a huge amount of labeled data. In most real-life scenarios, there is a scarcity of large volumes of such data which creates a bottleneck. This shortcoming has been addressed by using unsupervised learning. The general approach to this method uses a CNN feature extractor to extract meaningful information or interesting regions. Afterward, some clustering algorithm is used to cluster normal and anomaly samples separately (Doshi and Yilmaz, 2020b; Li et al., 2021). Closely related to this group of solutions are semi-supervised methods. Ruff et al. (2019) and Demertzis et al. (2020) adopt semi-supervised solutions for solving anomaly detection problems.

An approach to address data scarcity is zero-shot, one-shot, and any-shot learning (Ravi and Larochelle, 2016; Snell et al., 2017; Sung et al., 2018; Doshi and Yilmaz, 2020a; Lu et al., 2020; Rivera et al., 2020). Generally, different types of augmentations are used in this type of setting. Besides, these types of approaches require pretraining and guiding the gradient of the model using a large amount of labeled data from similar or related problems. In our case, the volume of data is limited. Furthermore, the definition of the anomaly is different between our dataset and the publicly available large datasets. For this reason, the power of few-shot learning or any-shot learning cannot be leveraged in our case.

Finally, after analyzing all the possibilities, we discard deep learning based approaches. We have a limited amount of data, which is not sufficient to make the CNNs learn the necessary feature representation. On the other hand, we cannot depend solely on a domain-related hand-crafted feature extraction method as it provides poor generalization. For this reason, we use machine learning based models and feed them with processed hand-crafted features from a few past frames and the current frame. The model then makes a decision on whether the current frame is normal or anomalous.

## 3 Dataset

### 3.1 Content and challenges

The dataset contains 3 sets of videos with a total of 19 video clips with a duration of approximately 10 s. The videos are captured using the camera placed randomly at 30 cm-200 cm from the liquid transfer device. Both the horizontal and vertical angles for the camera viewpoint is selected randomly from 0-15°. These values are selected empirically such that the resulting videos capture the transfer procedure robustly while having sufficient distractors to challenge the detection algorithm. In each video, the overall environment remains constant except for two moving parts. The first moving part is the table upon which there is a container of liquid. There are two types of containers. The first one is a series of glass trays containing a matrix of beakers. These beakers can either contain a liquid or are empty. And the second type of container is a plastic tub. The objective is to transfer the liquid from the source container to the target container. The second moving part is a robot end effector which contains a series of pipettes aligned in a row. These pipettes act as the middleman in the transfer of liquid between the containers. The effector can move both horizontally and vertically. It moves horizontally to place itself above the correct position of the source/target container and moves vertically to interact with them. The interaction of pipettes with the container is simply filling up the pipettes from the source container or emptying the liquid from the pipettes into the target container.

The primary challenge is the limited size of the dataset. Overall, 19 videos are present; five videos without any kind of anomaly and other videos containing bottom out and clogged tip anomalies. Video-level normal and anomaly labels are available out of the box. There are no readily available image-level labels, but are created for experiments based on manual observation. Among anomaly labels, a bottom-out anomaly occurs when the tip of the pipette is fixed against the bottom of a beaker while aspirating, which creates a vacuum and thus the pipette cannot function properly. Also, a clogged tip anomaly can occur where tips of the pipettes are completely or partially clogged and thus liquid cannot be extracted into the pipette properly. Here in this study, we focus on the anomaly cases that result in a change in the liquid level, and consequently, reduce the effectiveness of the liquid transfer task. Additional types of anomalies, such as the movement of the robot end effector or unpredictable changes to the environment causing mechanical and manual problems, are deferred to future works to reduce the challenges of the research problem. Most of the current studies into anomaly detection deal with supervised deep learning methods but due to the limited amount of data samples, our experiments show supervised deep learning under-performs in this case. To address this shortcoming, we develop methods using hand-crafted features.

Another challenge of this dataset is to make the solution color-invariant. As the liquids contained in the beakers can have various characteristics, the solution has to be effective for a wide range of liquid colors and shades. As methodologies based on hand-crafted features or geometric analysis depend on various thresholding and environment assumptions, these variations should provide a challenge regarding the robustness of the methods. The most challenging case is when the liquid is transparent. Here, the difference between the background and the pipette contents becomes almost indiscernible to the point that even a human eye cannot identify whether the liquid is present in the pipette or not. Only the transparent case remained unsolved in our experimentation. We surmise that detecting transparent liquid is not possible without major hardware modifications such as enhanced lighting using external devices, hyper-spectral imaging or other augmentations to the setup. We leave these experiments for future endeavors and focus on the applicability of the current setup for this study. Out of the 19 video clips, 13 contain colored liquids and the rest contain transparent liquids which we refrained from using in the experiments. Furthermore, the pipettes’ volume capacity and liquid extraction speed are also some parameters that can vary.

Additional difficulties are introduced in the case where the container of liquid is a whole tub. When the pipettes are lowered into the tub, it creates a ripple in the liquid of the container. This ripple intersects the region of filled liquid in the pipettes. As a result, the situation gets troublesome for handcrafted methods as additional noise is introduced. Furthermore, here the camera position changes and the pipette region of interest is further away from the camera. The further the camera is, the more noise is introduced in the pipette region. The camera also sometimes auto-focuses or can have slightly irregular movements which might result in noisy frames.

### 3.2 Data processing and augmentation

**FIGURE 1:**
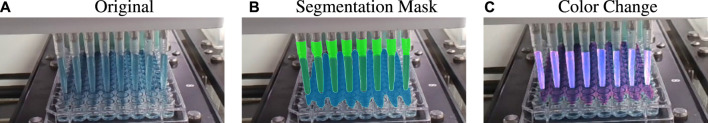
Example of liquid color augmentation. Here the original liquid color is cyan **(A)** and the manual segmentation masks **(B)** are used to change the color to violet **(C)**.

The video dataset is converted to an image-level dataset by frame extraction. The original video data has a frame rate of 30fps and each frame has a height and width of 720 and 1280, respectively. As the dataset contain only 19 video clips with limited variations of anomalies, an augmented dataset containing more videos and additional derived anomalous and normal cases will allow for more robust experiments. Thus, an additional augmented dataset is created using manual segmentation of the pipette shape and the liquid content of both the pipettes and the beakers present in a frame. By manipulating the pixels of the segmentation mask region, the brightness, contrast, and color of the original frames are modified to create new frame-level data as shown in Figure 1. The liquid color can vary depending on the experiment being performed in the laboratory but the dataset showcase some variations of this case, namely, cyan, red, green and yellow. Hence, such an augmentation is of paramount importance as this simulates various liquid color characteristics and can be used in both training and validation.

**FIGURE 2:**
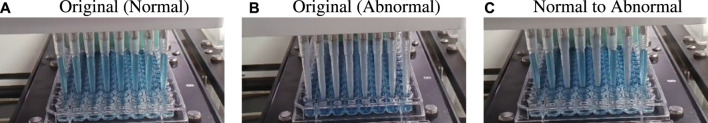
Example of pipette augmentation. Here the 3rd, 5th, and 7th pipettes of the normal frame **(A)** are replaced with those of an abnormal one **(B)** to produce a new abnormal frame **(C)**.

Another challenging aspect of the dataset is the case of pipettes with clogged tips. Only a few examples of this case are given, where some of the eight pipettes are clogged and cannot extract any liquid. However, this anomaly can affect any pipette in any order and in any number. That is why additional augmented data is created by utilizing the segmentations of the pipette shapes. It can be used to replace any segmentation of a normal case pipette with that of the abnormal case as shown in Figure 2. This pipette augmentation through segmentation is used to create abnormal case videos from normal videos and *vice versa*. Furthermore, the same type of augmentation is used on each frame of a single video in order to ensure video-level consistency. Otherwise, performing augmentation with different parameters on frames of a single video will violate the expected characteristics of natural videos.

For the manual segmentation, every 10th frame from the frame level dataset is considered. As the original dataset has a high frame rate, many subsequent frames are ignored due to minuscule changes. Furthermore, manual segmentation is a monotonous task, and segmenting each and every frame will cost valuable resources. Thus, only a subset of the relevant frame sequences is considered during the augmentation phase. The summary of the entire dataset is presented in Table 1.

**TABLE 1:** Dataset information.

Video source	Video class	Sample count
Laboratory	Normal	5
	Clogged	3
	Bottom Out	11
Segmentation	Normal	3
	Clogged	3

## 4 Methods and materials

Our methodology is inspired by the following observations. The anomaly scenarios can be detected more accurately during the liquid transfer phase between the container and the pipettes. During this phase, all the environmental elements remain static except the liquid inside the pipettes, which is moving up or down. Here, we denote the first frame of a video as *p*_1_ where a video has a total of *N* frames. By taking the difference between a frame *p*_*t*_ and subsequent ones *p*_*t*+*i*_ where *i* = 1, 2, ., *N*, this movement of liquid should be visible as it is the only dynamic object between the frames. If the number of detected liquid regions equals the number of pipettes and their movement corresponds to that of a working pipette, then the video can be classified as normal. Otherwise, the video is labeled as anomalous. Furthermore, the group of pipettes is the only region of interest that is a small part of the whole frame. In order to reduce computational complexity and eliminate noise and artifacts from the irrelevant space when comparing frames, we need a way to detect and crop this region of interest. In the next section, we describe the preprocessing developed to prepare the frames for the main processing task.

### 4.1 Preprocessing step

In order to detect the group of pipettes, the YOLOv5 object detection algorithm (Jocher et al., 2022) is employed. YOLOv5 is a one-stage detection algorithm that uses CSPDarknet53 with a Spatial Pyramid Pooling layer as the backbone, a Path Aggregation Network as the neck and a head from the original YOLO algorithm (Redmon et al., 2016). The algorithm outputs boundary box information of detected objects from a frame. The pipette region boundary boxes of selected frames from the original and augmented dataset are manually labeled and used for training, validation, and testing. The default parameters of the official YOLOv5 implementation (release v6.1) are used to fine-tune the model for 200 epochs. Here, as the model is already pretrained on the COCO dataset (Lin et al., 2014), the detection of the pipette region is a much easier task compared to anomaly detection which is evident by the fine-tuning results. The mean average precision score (map @ 0.5-0.95) of validation and testing is 0.916 and 0.928, respectively.

**FIGURE 3:**
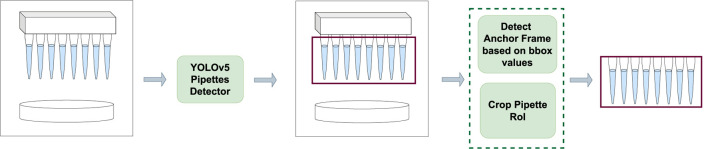
Self Comparison Preprocessing step. An input frame is passed through a fine-tuned YOLOv5 algorithm which detects the pipette region of interest. The detected boundary box values of each frame are used to detect the anchor frame and then the frame is cropped to the pipette region and passed on to the self comparison step.

The trained model is used to detect the pipette region from an incoming video stream. The region of interest is only useful when the pipettes are on top of the liquid container and have started interacting with the liquid. We call this frame the anchor frame *p*_*a*_. In order to identify this frame, the *y*-axis values of the detected boundary box from the model are extracted and compared. When the detected boundary box has moved downwards in the video stream and has stayed like that for 10+ frames, then the *p*_*a*_ frame is detected. The subsequent frames are cropped to the detected boundary box size and considered as the region of interest (RoI) for the next step. To account for noise and environmental changes, the boundary boxes from the algorithm are made 10% bigger. The preprocessing step is visualized in Figure 3.

### 4.2 Self-comparison step

After the preprocessing step, the incoming RoIs are compared with the detected anchor frame *p*_*a*_. The difference between the frames reveal the change of the pipette contents as time goes on which is used to classify the video as normal or as an anomaly. Let this difference frame be called Δ*p*. Environmental disturbances such as sudden irregular movements of the camera or the ripples of the liquid in the container during the transfer process can cause noise and artifacts in Δ*p*. To alleviate this problem, morphological operations are applied before further processing. For this task, the opening operation is used which is a sequence of erosion and dilation operations (Raid et al., 2014). The erosion operation uses a structuring element for reducing boundary shapes contained in the input image whereas the dilation operation is used to expand these shapes. By sequentially applying these operations, small noisy objects from the foreground of an image can be removed (Soille, 1999).

**FIGURE 4:**
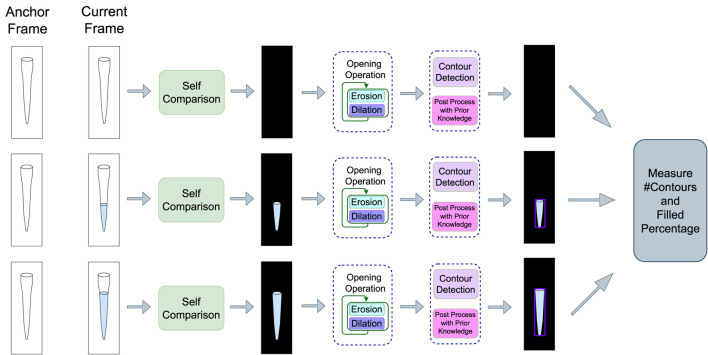
Self Comparison Method (single pipette view). Here, incoming frames are compared with the anchor frame to get the difference frames. The morphological opening operation is applied to them before contour detection. The detected contours are further post-processed using prior knowledge. The algorithm takes this output and extracts three types of features which is passed onto a predictor model for the final classification as an anomaly or normal frame.

Most of the noise and artifacts from Δ*p* are stripped away after applying the opening morphological operation. Then contours are detected from the cleaned RoI which point to the boundary of each liquid movement. This process is showcased in Figure 4. The size of the contours is supposed to increase on the *y*-axis if the pipettes are working correctly and the liquid is being successfully extracted. These contours are further filtered using prior knowledge about the environment and RoI. Firstly, the number of pipettes, *n*(*p*) is known beforehand, and thus the total number of contours should be the same. The detected contours are sorted by area and the largest *n*(*p*) contours are further processed. Secondly, each contour must have a minimum height to width ratio as its shape must conform to that of the pipette. Thirdly, these contours must always have vertical growth as that would mean the liquid level is rising in the pipette. All the relevant contours are filtered using these post-processes and tracked.

### 4.3 Median width denoising

Due to noise present in the difference frame Δ*p*, some of the detected and post-processed contours’ widths might have some discrepancy with the pipettes’. In order to alleviate this deviation, the prior knowledge that the width of all the pipettes is the same is used. As most of the noise is stripped away due to the aforementioned operations, most of the contours portray accurate width information of the pipettes. That is why the median of all the detected contours’ widths can be a good estimate of the actual width and is used instead of the original ones. The algorithm outputs the bounding boxes of the contours with the width set as this median value. Thus, the width discrepancy can be avoided and denoised using median width.

### 4.4 Features for learning

We extract three types of features from the previous step and pass into a final predictor in order to detect soft scores for anomaly detection. The first type is the boundary box information of the contours for each detected pipette for each frame. The boxes can also give approximate information on how much a pipette has been filled with a liquid. The height and width of a detected contour box gives its area, *area*_*contour*_. Furthermore, the original pipette area, *area*_*pipette*_, can be estimated approximately by taking the original RoI bounding box’s height and a width and comparing it with the number of pipettes. By taking the ratio of the square root values of *area*_*contour*_ and *area*_*pipette*_, we can get an approximation of how much a pipette is filled which is the second type of feature we extract. This feature contains notable information to deduce whether a pipette is working properly or not and thus this feature is also taken into consideration. Nevertheless, the previous information provides an incomplete picture of the current state of the pipette if only the current frame is being considered. In order to provide temporal context to the current pipette’s state, previous frame pipette information or some form of *history* is also needed. Hence, the detected boundary boxes and ratio information of previous frames are also utilized as the third type of feature for the final prediction stage. Finally, the predictor model outputs a probabilistic score on whether the current frame features constitute a normal or an anomalous event. Later on in Section 5.1, we conduct ablation studies on the type of the final predictor and demonstrate that Support Vector Classifier (SVC) provides the best performance for this task.

## 5 Experiments

In order to quantify the performance of the proposed method and compare with other methodologies, we compute ROC curve and use Area Under the Curve (AUC) and Equal Error Rate (EER) metrics. Here in Section 5.1, we provide comparison of results from different machine learning classifiers. Afterward, in Section 5.2, we compare the best performing predictor in our model with other baselines for video anomaly detection including deep learning methods. Finally, the effect of different components of our methods are discussed in Section 5.3.

### 5.1 Comparison of different machine learning classifiers

**FIGURE 5:**
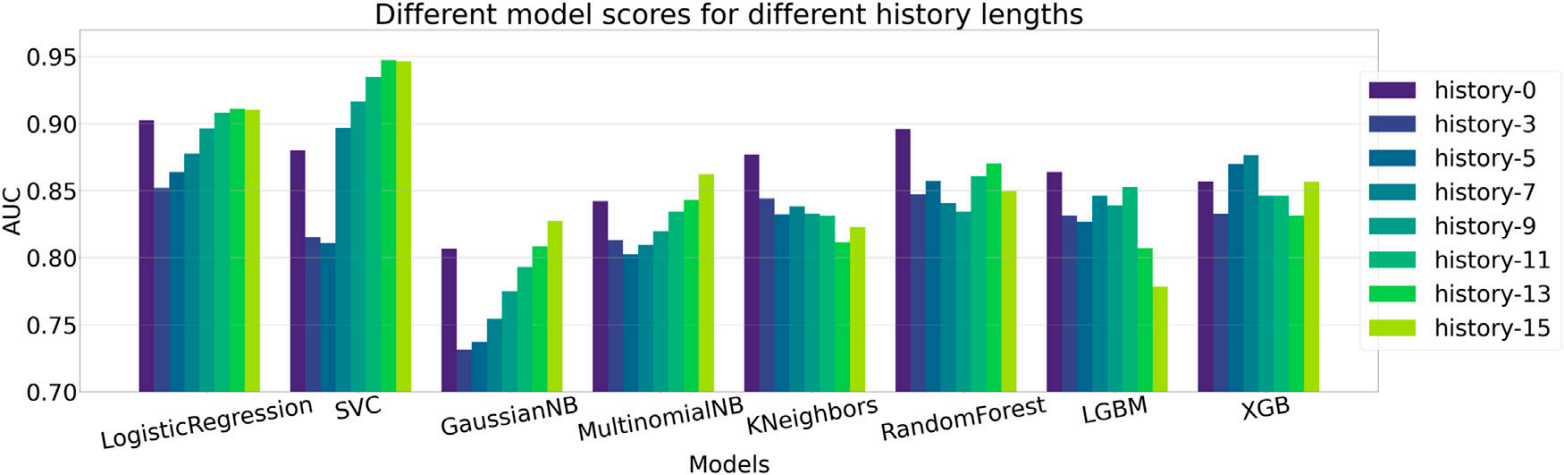
Comparison of AUC scores between different ML methods. Logistic Regression, SVC, GaussianNB, MultinomialNB and XGB show gradual improvement over their baseline score when history is introduced. Here SVC scores the most with an AUC of 94.74% in the history-13 setting.

**FIGURE 6:**
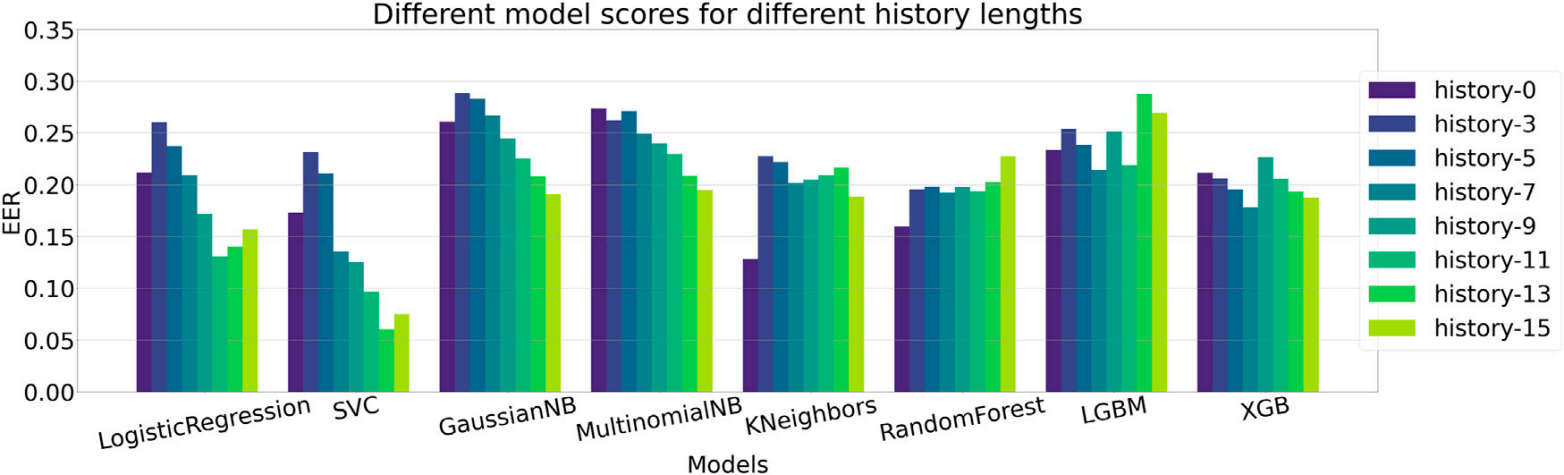
Comparison of Equal Error rate scores between different ML methods. Logistic Regression, SVC, GaussianNB, MultinomialNB and XGB show gradual improvement over their baseline EER score when history is introduced, similar to the AUC score scenario in Figure 5. Here, SVC has the least EER of 6.06% in the history-13 setting, making it the best performing model.

We evaluate the performance of different machine learning models to identify the one that provides the best prediction, given the features extracted via the self comparison method in Section 4.4. We compare the performance among different methods and also evaluate the impact of providing previous *n* frames information along with the current frame, a setting we define as *history*. A complete ablation study of AUC scores on different history lengths for different machine learning models are given in Table 2 and the same is given for EER scores in Table 3. The bold values correspond to best performing model for each history and the underlined values show the best performance for each of the models. When there is no history, i.e., only the current frame history is present, Logistic Regression scores the most in terms of AUC with a score of 90.23% where Support Vector Classifier (SVC) scores 87.99%. In terms of no-history EER, KNeighbors has the best score of 12.82% where SVC scores 17.31%. When history length of 3 is added, the scores of all the models fall, which may point to insufficient temporal context. This explains why as more history or temporal context is added, five of the eight tested models show improvement over their initial no-history AUC score and six of the eight models show improvement in terms of EER. Figure 5 contains the barplots of the AUC scores; Figure 6 shows the barplots of EER (Equal Error Rate) scores and they both paint a similar picture in regards to the models performances. The methods KNeighbors and RandomForest show no improvement in both metrics when any amount of history is added which may point to their inability to capture the additional context provided by previous frame information. XGBoost shows improvement up-to history-7 and then the score decreases signifying that there might be an optimal history length for each of the models. The SVC model has the overall best score when history length of 13 is applied, with an AUC of 94.74% which is 7% more than its no-history AUC and an EER of 6.06% which is 64.99% better than its no history EER. Though SVC is outperformed by Logistic Regression in terms of AUC and KNeightbors in terms of EER in the no history case, it manages to surpass their scores comfortably when history is applied. Furthermore, the SVC model scores the best across these 2 metrics among all the classifiers apart from the no-history, history-3 and history-5 cases. Thus, adding history frame information helps performance by providing historical context and the SVC method consistently provides the best score as more context is given. Due to these two observations, the SVC classifier in addition to a history length of 13, is chosen as the predictor for the final pipeline.

**TABLE 2:** AUC score (in percentage) comparison of different machine learning models over different history lengths. History length refers to how many previous frames’ pipette features are used; e.g., history-13 means that previous 13 frames’ features are used alongside the current frame features. The bold values show best performance for each history. The underlined values show the best performance for each model.

Models	No history	History-3	History-5	History-7	History-9	History-11	History-13	History-15
Logistic Regression	**90.23**	**85.21**	86.40	87.73	89.62	90.79	91.10	91.00
Support Vector Classifier	87.99	81.51	81.09	**89.66**	**91.62**	**93.46**	**94.74**	**94.64**
Gaussian Naive Bayes	80.66	73.12	73.71	75.41	77.48	79.29	80.82	82.73
Multinomial Naive Bayes	84.21	81.29	80.24	80.95	81.96	83.41	84.31	86.23
KNeighbors	87.68	84.41	83.21	83.80	83.25	83.10	81.16	82.27
RandomForest	89.56	84.73	85.73	84.06	83.41	86.06	87.02	84.97
LightGBM	86.38	83.14	82.66	84.61	83.88	85.24	80.71	77.82
XGBoost	85.67	83.27	**86.96**	87.63	84.59	84.60	83.14	85.64

**TABLE 3:** EER score (in percentage) comparison of different machine learning models over different history lengths. History length refers to how many previous frame’s pipette features are used; e.g., history-13 means that previous 13 frames’ features are used alongside the current frame features. The bold values show best performance for each history. The underlined values show the best performance for each model.

Models	No history	History-3	History-5	History-7	History-9	History-11	History-13	History-15
Logistic Regression	21.19	26.04	23.73	20.92	17.17	13.07	14.01	15.67
Support Vector Classifier	17.31	23.14	21.07	13.51	12.53	9.61	6.06	7.50
Gaussian Naive Bayes	26.08	28.85	28.30	26.66	24.47	22.50	20.80	19.08
Multinomial Naive Bayes	27.35	26.21	27.11	24.92	23.98	22.95	20.87	19.46
KNeighbors	12.82	22.71	22.17	20.18	20.48	20.93	21.65	18.86
RandomForest	15.95	19.53	19.80	19.22	19.77	19.37	20.24	22.73
LightGBM	23.35	25.36	23.82	21.42	25.10	21.90	28.75	26.92
XGBoost	21.14	20.59	19.55	17.84	22.66	20.55	19.33	18.72

### 5.2 Comparison with different methods

We compare our developed method with existing methodologies proving our method’s effectiveness for this dataset. As the dataset does not contain an adequate number of labeled data, we experiment with a few-shot learning method (Huang et al., 2022). This method uses image registration as a proxy task to learn the distribution of non-anomaly samples during training time and was pretrained on MVTec (Bergmann et al., 2019) dataset. The AUC score for this method is 52.37%, and the EER is the largest among the tested methods. This performance can be attributed to the huge difference in characteristics between the pretraining dataset and our dataset. Furthermore, as the dataset contains ideo-level labels, we use the deep multiple instance ranking framework (Sultani et al., 2018) method to get a detection baseline score. This method performs slightly better than the Few Shot Learning method, but still the method cannot give a usable score due to its dependency on high number of training samples. Moreover, to test a baseline self-supervised method for this task, we adopt the video anomaly detection paper (Wang G. et al., 2022), which tries to detect anomalies by solving decoupled spatio-temporal jigsaw puzzles. A model pretrained on the Shanghai Tech dataset is used but it results in an AUC score of 62.30%, which maybe be due to the characteristic differences between the datasets.

Finally, we try frame-level binary classification with ResNet architectures (He et al., 2016). The default setting uses a single frame as input with three channels (RGB). To make this a fair comparison with the machine learning methods, the cropped outputs of the preprocessed steps are used. This yields an AUC of 77.16% and an EER of 26.29% which is the best among all the previous methods. Similar to the machine learning comparisons, we try incorporating previous frame information with the input by stacking the previous 15 consecutive frames along with the current frame on the channel dimension. But here, the AUC score drop about 3%–75%. This score can be attributed to the fact that as we are incorporating the previous 15 frames our training dataset should also be increased 15*X* to maintain the same rate of information. Furthermore, 2D convolutions cannot preserve the temporal context of the frame stack which can decrease the score. This provides the rationale for further experimentation with 3D convolutions via 3D Resnet using the previous settings. Here the AUC score is 79.55%, which is an improvement of 3% compared to the 2D Resnet score, showing that the 3D version can gain some temporal contextual information.

**FIGURE 7:**
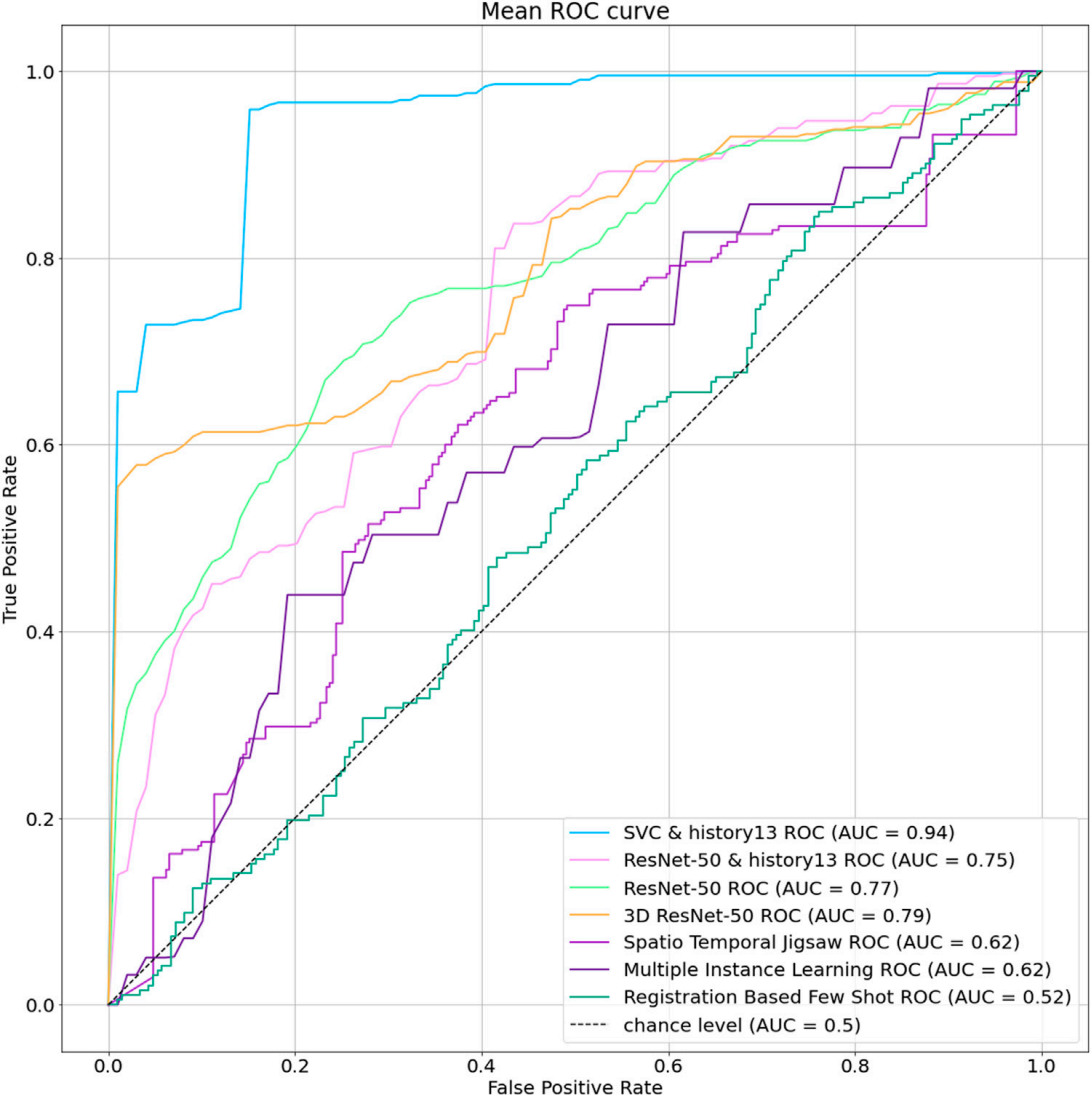
ROC Curve comparison between our developed method and existing anomaly detection models.

Our developed methodology beat all the experiments by a large margin. The 3D Resnet model performs the best among all the deep learning methods with an AUC score of 79.55% and an EER of 25.26%. On the other hand, our method has an AUC of 94.74% and an EER of 6.06% which is a 19% AUC and 76% EER improvement. Thus, our developed method provides the most reliable outputs among all the existing methodologies. A comparison between the stated methods with our developed method is presented in Table 4. Additionally, the ROC Curve comparison between the methods is shown graphically in Figure 7.

**TABLE 4:** AUC and EER Score comparison of best-performing machine learning model with existing anomaly detection methods of different kinds. 3D ResNet-50 scored the best among existing deep learning methods. Our developed method with a SVC predictor outperforms all across both metrics.

Method	AUC	EER
Registration Based Few-Shot Anomaly Detection	52.37	47.38
Deep Multiple Instance Learning	62.04	43.01
Decoupled Spatio-Temporal Jigsaw Puzzle	62.30	37.66
ResNet-50	77.16	26.29
ResNet-50 with history-15	75.00	26.77
3D ResNet-50	79.55	25.26
Support Vector Classifier with history-13	94.74	6.06

All the aforementioned results are produced for the colored liquid transfer task. When we incorporate the examples of transparent liquids, the SVC model AUC score drops to 77% and the Resnet AUC score drops below 50%. In the event the liquid is transparent, the self-comparison method cannot correctly capture the contrast between the liquid filled pipette and the empty pipette. Thus, the output features of this method become very noisy and the SVC model’s predictions become skewed towards anomaly even in the presence of a normal example. Furthermore, only one normal sample of transparent liquid transfer event is present and thus there cannot be an effective cross validation for this special case which is one of the main reasons deep learning methods are ineffective here. Consequently, the difficult problem of transparent liquid transfer remains unsolved in our case, where data scarcity and imbalance played a big role in the outcome.

### 5.3 Output result analysis

Here, we compare the effect of some components of the developed method. Mainly the results of excluding and incorporating the opening operation and post-processing operations in the developed methodology are discussed. Then, the output of the self-comparison method is shown which is used to output the filled percentage of liquid in the pipettes as auxiliary information.

### 5.3.1 Effect of opening operation

**FIGURE 8:**
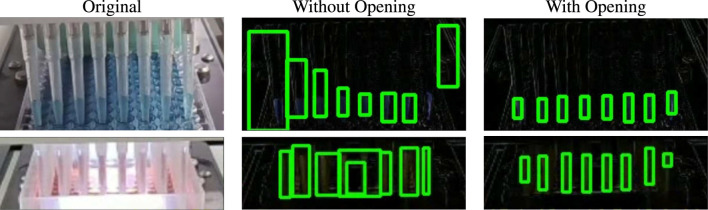
Effect of morphological operation. The first row depicts the case when sudden camera movement caused noise to appear in Δ*p*. The second row shows what happens when water ripples in the tray effect the contour detection. Both are resolved using the opening operation.

The opening morphological operation protects against noise and makes the solution robust against rapid irrelevant environmental change. For example, the camera viewing the pipettes can sometimes shake or lose focus, and it then immediately re-adjusts. But the frames which show this instantaneous change have the difference frame Δ*p* filled with irrelevant artifacts. The opening operation removes this noise from the frame and stabilizes Δ*p*. This case is shown in the top row of Figure 8.

Another example of noise reduction through this operation is the case showcased in the bottom row of Figure 8. Here, the tray is filled with liquid and the pipettes extract the contents directly from it. This can cause a ripple effect due to the movement of the liquid which is visible in Δ*p*. But this information is irrelevant to the pipette contours and causes additional noise. The opening operation once again gets rid of this noise and outputs only the relevant portion of Δ*p* before contour detection.

### 5.3.2 Effect of prior knowledge post-process

**FIGURE 9:**
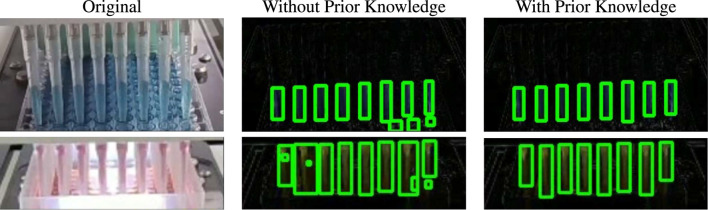
Effect of post-processing operation. In both cases, there are small noisy contours with abnormal height by width ratios which are discarded after post-processing using prior knowledge.

The post-processing operations utilize prior knowledge to filter the detected contours and output only the relevant ones. The opening operation cannot remove all possible noise and artifacts and thus post processing plays a huge role in identifying the useful contours. As the prior knowledge during this step will remain constant throughout the life cycle of the experiments, it can be used to effectively extract the contours best matching with the pipettes. The images in Figure 9 show how the unprocessed images have few noisy contour boxes with a comparatively negligible area or whose width is greater than the height. These objects are also irrelevant to the pipette liquid shape and are filtered out to compute the final contours which are then reshaped using median width denoising. Then, relevant information like the number of contours and pipette filled percentage is used to identify whether the video frames represent a normal procedure or not.

### 5.3.3 Final output

**FIGURE 10:**
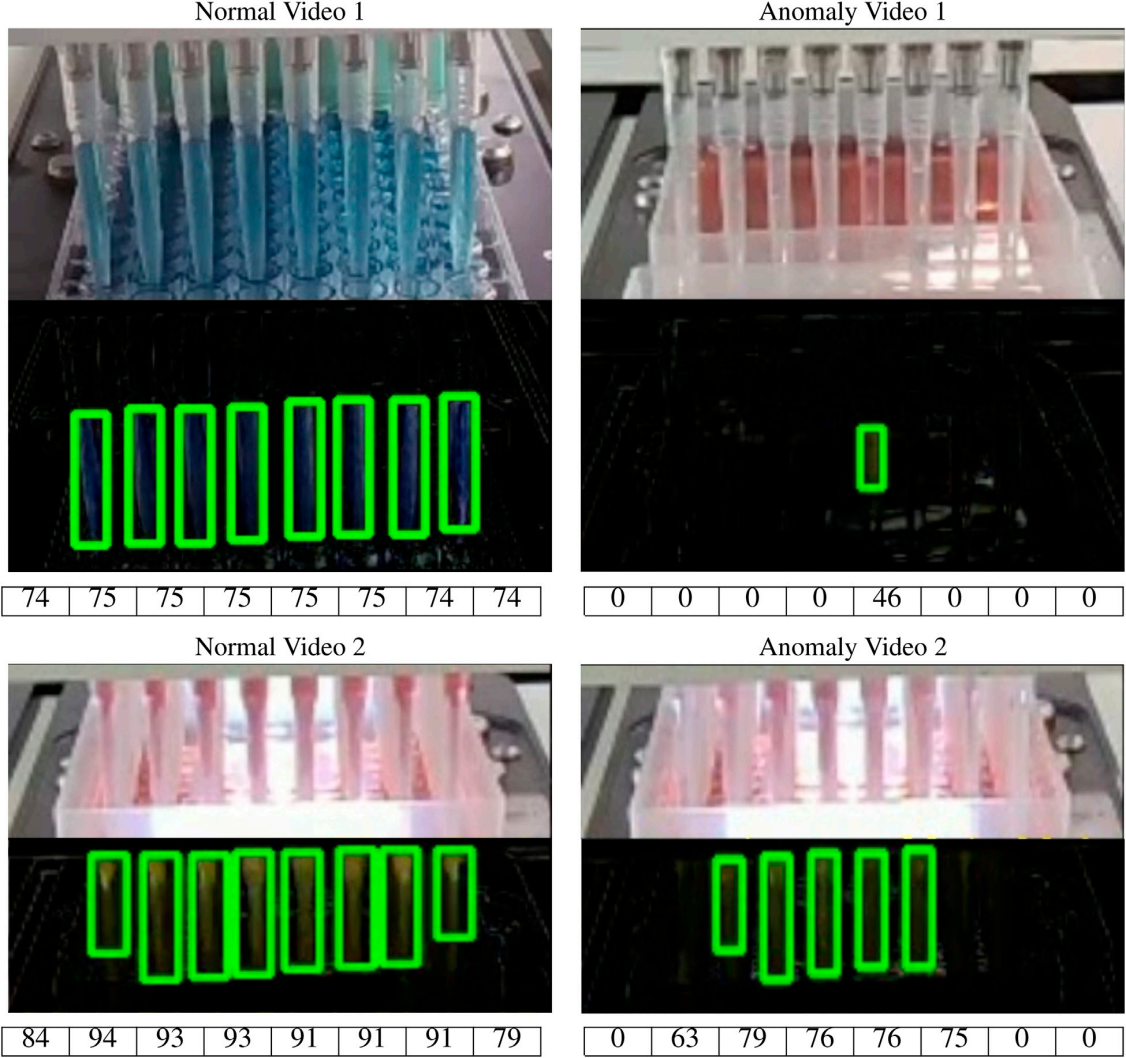
Results of different video frames with each pipette filled percentage shown in a table below each video frame. The first column contains normal videos as the number of detected contours is equal to the number of pipettes and over 50% of each pipette is filled with liquid. The 2nd column features anomaly cases where the number of detected contours didn’t match the number of pipettes.

The method is able to successfully differentiate between the normal and anomalous videos in the dataset which have colored liquids. Figure 10 shows the decisive Δ*p* frames and the extracted contours of some of the video cases. The approximate pipette-filled percentage using area ratio approximation is also shown below each final contour output. The first column of the figure contains normal examples where the number of pipettes equals the number of contours detected and more than 50% of each pipette is filled up. In the second column, examples containing anomaly are shown. In Anomaly Video 1, only a single pipette is detected and it isn’t filled up to even 50%. Thus, it is classified as an anomaly. In Anomaly Video 2, all the pipettes have more than 50% filled up. However, the total number of contours did not match the total number of pipettes. Therefore, this frame is classified as an anomaly. In these cases, we consider 50% as the minimum level for which a pipette is considered as an anomaly. But this threshold value should be different based on the experiment environment specifications like the camera-setup distance and the task requirements. Thus, this value should be tuned based on the desired false acceptance rate of the algorithm and also the provisions of the target task.

## 6 Conclusion

Automatic anomaly detection can undoubtedly save hours of human labor and is much needed in automated laboratory procedures where anomalies could result in faulty conjectures or failed experiments. If an anomaly can be correctly detected in this scenario, steps to alert, diagnose, and auto-correct the procedure can be initiated. Here, we presented a novel dataset for video anomaly detection in laboratory setups with the task of liquid transfer. The dataset introduces several challenges, such as limited number of samples, ripples in the liquid container, and varying environmental conditions.

Because of data scarcity and variable environmental conditions, conventional deep learning models cannot provide a satisfactory result. As a result, we presented a feature-based method to address the several challenges that might occur in such scenarios. The proposed method is color-invariant and provides high accuracy despite the aforementioned challenges. Several experiments and ablation studies confirm the effectiveness of the proposed method. In particular, the proposed method surpasses the state-of-the-art methods of anomaly detection by 19% and achieves 94.74% AUC in detecting anomalous events.

## Data Availability

The datasets presented in this article are not readily available because The dataset is currently private since the data acquisition is still in progress. However, the dataset will be released later on when the procedure is done. Requests to access the datasets should be directed to xumin100@gmail.com.
